# Prescribing patterns and determinants for elderly patients with Parkinson's disease in Japan: a retrospective observational study using insurance claims databases

**DOI:** 10.3389/fneur.2023.1162016

**Published:** 2023-06-23

**Authors:** Morinobu Seki, Yayoi Kawata, Ayako Hayashi, Masaki Arai, Shinji Fujimoto

**Affiliations:** ^1^Department of Neurology, Keio University School of Medicine, Tokyo, Japan; ^2^Japan Medical Office, Takeda Pharmaceutical Company Limited, Tokyo, Japan

**Keywords:** aged, Japan, longitudinal studies, Parkinson's disease, prescribing pattern, elderly

## Abstract

**Background:**

This study aimed to determine real-world prescribing patterns and determinants for Japanese patients with Parkinson's disease (PD), with a focus on patients ≥75 years.

**Methods:**

This was a retrospective, observational, longitudinal study of patients with PD (≥30 years, ICD-10: G20 excluding Parkinson's syndrome) from three Japanese nationwide healthcare claim databases. Prescription drugs were tabulated using database receipt codes. Changes in treatment patterns were analyzed using network analysis. Factors associated with prescribing patterns and prescription duration were analyzed using multivariable analysis.

**Results:**

Of 18 million insured people, 39,731 patients were eligible for inclusion (≥75-year group: 29,130; <75-year group: 10,601). PD prevalence was 1.21/100 people ≥75 years. Levodopa was the most commonly prescribed anti-PD drug (total: 85.4%; ≥75 years: 88.3%). Network analysis of prescribing patterns showed that most elderly patients switched from levodopa monotherapy to adjunct prescription patterns, as did younger patients, but with less complexity. Elderly patients who newly initiated PD treatment remained on levodopa monotherapy longer than younger patients; factors significantly associated with levodopa prescriptions were older age and cognitive impairment. Commonly prescribed adjunct therapies were monoamine oxidase type B inhibitors, non-ergot dopamine agonists, and zonisamide, regardless of age. Droxidopa and amantadine were prescribed as adjunct levodopa therapy slightly more frequently among elderly patients; levodopa adjunct therapy was prescribed when the levodopa dose was 300 mg, regardless of age.

**Conclusion:**

Prescribing patterns for patients ≥75 years were levodopa centered and less complex than for those <75 years. Factors significantly associated with levodopa monotherapy and continued use of levodopa were older age and cognitive disorder.

**Clinical trial registration:**

UMIN Clinical Trials Registry, https://center6.umin.ac.jp/cgi-open-bin/ctr_e/ctr_view.cgi?recptno=R000053425 (UMIN000046823).

## 1. Introduction

Parkinson's disease (PD) is a neurodegenerative disorder characterized by slow, progressive motor dysfunction that mainly affects individuals aged >65 years (1–3). Because the number and proportion of people ≥65 years are rapidly increasing worldwide, the number of people with PD is estimated to cross 12 million by 2040 (1). In Japan, where the aged population is increasing at a record rate, the number of people with PD has increased 2-fold from 1987 to 2017, and the proportion of people with PD aged ≥75 years is estimated to account for 64.8% of all people with PD (4, 5). An aged population with PD has significant consequences for long-term care, particularly in terms of increasing frailty, polypharmacy, comorbidities, and adverse drug effects (6, 7).

At present, pharmacotherapy for PD is based on the management of both motor and non-motor symptoms (8) with multiple anti-PD drugs. Levodopa is most commonly prescribed for the initial treatment of motor symptoms (9), and levodopa-induced complications, including wearing-off and dyskinesia, are managed by adjusting the dosage, administering levodopa, or adding other drugs such as dopamine agonists (DAs), catechol-*O*-methyltransferase (COMT) inhibitors, or monoamine oxidase type B (MAO-B) inhibitors (8). Non–anti-PD drugs are also used for a variety of non-motor symptoms such as constipation, urinary frequency, pain, insomnia, hallucination, and cognitive impairment (10). The pharmacological management of patients with PD is complex and determined by multiple factors (9). Although age is the most common patient-related factor that determines an anti-PD drug prescription (9), elderly patients with PD are excluded from many clinical studies because of their severe PD symptoms and comorbidities. Consequently, there is little evidence-based information to support treatment for these patients, and the current status of how these patients are treated is unclear. The Japanese treatment guidelines (11) recommend that elderly patients are mainly treated with levodopa because of the risk of developing psychiatric symptoms but provide little other guidance for these patients.

All Japanese citizens have guaranteed public health insurance with free access to the medical institutions of their choice and receive advanced medical care at a low cost (approximately 10–30% of total medical costs). Several studies have been conducted to assess real-world prescribing patterns for patients with PD in Japan (12–14). Data from these studies were extracted from single nationwide medical claims databases with limited coverage for patients ≥75 years (15). With the implementation of the elderly healthcare system in 2008, people aged ≥75 years are now covered by this system, and the elderly health insurance database has become available for research use (DeSC Healthcare, Inc., Tokyo, Japan). The objectives of this study were to determine how real-world PD prescribing patterns change with age and to assess factors associated with prescribing patterns, with a focus on elderly patients ≥75 years, using claims data from three nationwide databases that provide coverage for elderly patients, for people who are self-employed or unemployed, and for company employees.

## 2. Materials and methods

### 2.1. Study design

This was a retrospective observational study (data extraction period: from June 2016 to May 2021) conducted using healthcare claims data extracted from three nationwide databases (15) in Japan: (1) the elderly health insurance database (hereinafter Elderly database; DeSC Healthcare, Inc., Tokyo, Japan), a medical insurance system for people ≥75 years in which 2.6 million people are registered; (2) the JMDC database (JMDC Inc., Tokyo, Japan), a medical insurance system for company employees and their dependents (< 75 years) in which 13.2 million people are registered; and (3) the National Health Insurance (NHI) database (DeSC Healthcare, Inc., Tokyo, Japan), a medical insurance system for people < 75 years who are not eligible for the Elderly database or the JMDC database (e.g., the self-employed) in which 2.3 million people are registered.

**Figure 1:**
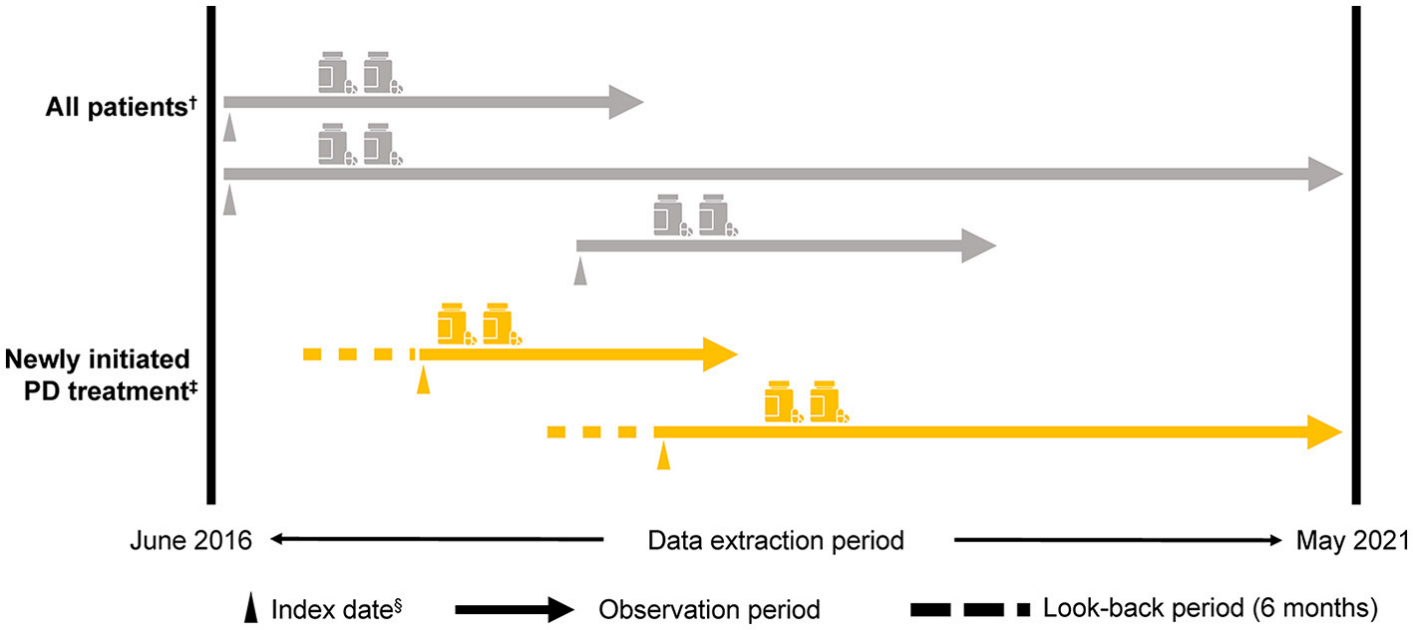
Study design. ^†^≥30 years, a diagnosis of PD (i.e., an ICD-10: G20 [excluding Parkinson's syndrome] claim) in the observation period and for ≥6 months from the index date, at least one anti-PD drug prescription for ≥6 months from the index date, and at least two anti-PD drug prescriptions during the observation period that were ≥6 months from the index date. ^‡^Patients who newly initiated PD treatment (i.e., a first ICD-10: G20 claim for an anti-PD drug prescription) within the observation period ≥6 months from the start of the observation period to the index date. Medical history and/or comorbidity record was evaluated 3 months before the index date. ^§^The first day of the month in which the first ICD-10: G20 claim was recorded. ICD-10, *International Classification of Diseases and Related Health Problems, Tenth Revision*; PD, Parkinson's disease.

The index date was the first day of the month that a patient was assigned a claim for PD, excluding Parkinson's syndrome (i.e., an *International Classification of Diseases and Related Health Problems, Tenth Revision* [ICD-10] code G20 claim) during the observation period. Patients aged ≥30 and < 75 years from the NHI and JMDC databases were included in the < 75-year group, and patients from the Elderly database were included in the ≥75-year group (Supplementary Figure 1). The anti-PD drugs approved in Japan (Supplementary Table 1) were evaluated using the corresponding Anatomical Therapeutic Chemical (ATC) codes. Patients were tracked from the index date until removal from the database for any reason (e.g., changed insurance system and death) or until the end of the observation period (Figure 1).

The study protocol was approved by the Research Institute of Healthcare Data Science Institutional Review Board (No. RI2021024) in Japan. Data were collected and analyzed in accordance with the Japanese Ethical Guidelines for Medical and Health Research Involving Human Subjects (16); in accordance with these guidelines, informed consent was not required because only anonymized information was accessible from each database. The study was registered at UMIN Clinical Trials Registry [https://center6.umin.ac.jp/cgi-open-bin/ctr_e/ctr_view.cgi?recptno=R000053425 (UMIN000046823)].

### 2.2. Study population

Patients were included in the analyses (All patients) if they were aged ≥30 years, were assigned an ICD-10 code for PD (G20) at the index date, had a PD record ≥6 months from the index date, had a prescription for at least one type of anti-PD drug, and had at least two prescriptions of any anti-PD drug type issued during the observation period (Figure 1).

Patients who newly initiated PD treatment were categorized as having PD for the first time (newly initiated PD treatment patients). This category was based on patients who did not have an anti-PD drug prescription for ≥6 months from the start of the observation period to the index date. Patients with ICD-10 codes related to drug-induced Parkinson's syndrome or cerebrovascular parkinsonism (13) up to 6 months before the index date were excluded.

### 2.3. Outcome measures

Outcome measures included the prevalence of PD, database characteristics, patient demographics and clinical characteristics, and comorbidities, which were obtained from the diagnosis code (Supplementary Table 2). Anti-PD drugs were defined as those drugs prescribed for the treatment of PD during the observation period. Code lists for comorbidities and the Charlson Comorbidity Index (CCI) were based on updated versions revised specifically for ICD-10 insurance claims data (17). A time frame of up to 6 months before the index date was used to screen for the presence/absence of each comorbidity, and the CCI was calculated as the weighted sum of present comorbidities. For prescribing patterns, a summary of anti-PD drugs prescribed since the index date was compiled, and prescription combinations were identified. Treatment patterns were defined as prescription combinations that continued for ≥7 consecutive days. The prescription period was calculated based on the number of scheduled administration days associated with each prescription. If there was an additional prescription within 90 days of the last day of the prescription period, the anti-PD drug was considered to have been continued, including the blank period. If there was no additional prescription within 90 days, the drug prescription was considered to have ended on the last day. Levodopa dosage was calculated using the daily prescribed dose of levodopa based on the prescription receipt. Patients receiving deep brain stimulation or using levodopa-carbidopa intestinal gel were excluded from the levodopa dosage calculation.

### 2.4. Statistical analysis

Data from all three databases were integrated for the analysis of All patients. For continuous variables, mean and standard deviation or median and 25th/75th percentiles were calculated. In All patients, a graphical representation of the changes in treatment patterns over time was constructed using a network diagram comprising nodes and edges regardless of the point of the first or second prescribing pattern. The change in levodopa dose at the time of the second anti-PD drug prescription was summarized using a histogram and box plot. The factors associated with prescribing patterns were analyzed by logistic regression, and drug duration was analyzed by Cox proportional hazard regression analysis. Possible multicollinearity between the explanatory variables and variable selection was taken into consideration beforehand. After binary classification (e.g., levodopa vs. other), point estimates, odds ratios, and 95% confidence intervals for each factor were reported. The effect of prescribing patterns on drug duration was analyzed using multivariable analysis, and the Kaplan–Meier method was used to plot survival curves of duration from the first to the second treatment pattern in patients who newly initiated PD treatment. To compare demographics between the ≥75-year and < 75-year groups, the Wilcoxon rank sum test for continuous variables and the chi-square test for categorical variables were used. Fisher's exact test was used when the expected frequencies were small; differences with a *P* < 0.05 were considered statistically significant. All analyses were conducted using SAS 9.4 (SAS Institute, Cary, NC, USA) and R, Version 4.1.1 (R Foundation for Statistical Computing, Vienna, Austria).

## 3. Results

### 3.1. Study population

Of the 18 million insured people across all three databases, 39,731 were eligible for inclusion: 29,130 in the ≥75-year group and 10,601 in the < 75-year group (Supplementary Figure 1). Of these 3,468 were categorized as newly initiated PD treatment patients (1,791 ≥75 years and 1,677 < 75 years). The prevalence of PD between 2020 and 2021 among All patients in the ≥75-year group was 1.21% (Supplementary Table 3). The mean observation time (data acquisition period) was 2.6 years and 3.6 years for the ≥75-year and < 75-year groups, respectively (Table 1A).

**Table 1:** Demographics and clinical characteristics of patients with PD included in this analysis.

**A: All patients**				
**Characteristic**	**Total;** ***N*** = **39,731**	≥**75 years;** ***N*** = **29,130**	<**75 years;** ***N*** = **10,601**	**Group difference;** ***p*****-value**
Male, *n* (%)	17,007 (42.8)	11,552 (39.7)	5,455 (51.5)	0.000
Age (years)	75.8 ± 11.1	81.3 ± 5.0	60.9 ± 9.5	0.000
Age, *n* (%)				
≤ 54 years	2,322 (5.8)	NA	2,322 (21.9)	NA
55–64 years	3,401 (8.6)	NA	3,401 (32.1)	NA
65–74 years	4,878 (12.3)	NA	4,878 (46.0)	NA
75–84 years	21,713 (54.7)	21,713 (74.5)	NA	NA
≥85 years	7,417 (18.7)	7,417 (25.5)	NA	NA
Observation period (years)	2.8 ± 1.2	2.6 ± 1.0	3.6 ± 1.4	0.000
Duration of PD treatment (days)	799.9 ± 435.5	738.5 ± 380.9	968.5 ± 522.8	0.000
Charlson Comorbidity Index	3.5 ± 2.7	3.9 ± 2.7	2.4 ± 2.4	0.000
Number of anti-PD drug types	2.3 ± 1.4	2.2 ± 1.3	2.6 ± 1.6	0.000
Number of any drugs^†^, ATC main category	4.9 ± 2.1	4.9 ± 2.0	4.7 ± 2.2	0.000
Comorbidities, *n* (%)				
Constipation	32,909 (82.8)	25,712 (88.3)	7,197 (67.9)	0.000
Insomnia	21,670 (54.4)	16,491 (56.6)	5,116 (48.3)	0.000
Pain	20,777 (52.3)	16,703 (57.3)	4,074 (38.4)	0.000
Cognitive disorder	15,871 (39.9)	14,263 (49.0)	1,608 (15.2)	0.000
Depression and anxiety	14,195 (35.7)	10,250 (35.2)	3,945 (37.2)	0.000
Hallucination	2,114 (5.3)	1,759 (6.0)	355 (3.3)	0.000
**B: Patients who newly initiated PD treatment**				
**Characteristic**	**Total;** ***N*** = **3,468**	≥**75 years;** ***N*** = **1,791**	<**75 years;** ***N*** = **1,677**	***p*** **-value**
Male, *n* (%)	1,650 (47.6)	747 (41.7)	903 (53.8)	0.000
Age (years)	72.1 ± 12.0	81.4 ± 4.5	62.1 ± 9.0	0.000
Age, *n* (%)				
≤ 54 years	308 (8.9)	NA	308 (18.4)	NA
55–64 years	549 (15.8)	NA	549 (32.7)	NA
65–74 years	820 (23.6)	NA	820 (48.9)	NA
75–84 years	1,347 (38.8)	1,347 (75.2)	NA	NA
≥85 years	444 (12.8)	444 (24.8)	NA	NA
Observation period (years)	3.7 ± 1.0	3.2 ± 0.8	4.3 ± 0.9	0.000
Duration of PD treatment (days)	640.4 ± 345.8	566.0 ± 282.6	719.9 ± 387.2	0.000
Charlson Comorbidity Index^‡^	1.2 ± 1.7	1.6 ± 1.8	0.8 ± 1.4	0.000
Number of anti-PD drug types	1.8 ± 1.0	1.6 ± 0.8	2.1 ± 1.2	0.000
Number of any drugs^†^^, ‡^, ATC main category	2.3 ± 1.9	2.8 ± 1.9	1.9 ± 1.7	0.000
Comorbidities^‡^, *n* (%)				
Constipation	1,336 (38.5)	947 (52.9)	389 (23.2)	0.000
Insomnia	827 (23.8)	539 (30.1)	288 (17.2)	0.000
Pain	890 (25.7)	632 (35.3)	258 (15.4)	0.000
Cognitive disorder	300 (8.7)	264 (14.7)	36 (2.1)	0.000
Depression and anxiety	604 (17.4)	331 (18.5)	273 (16.3)	0.096
Hallucination	24 (0.7)	14 (0.8)	10 (0.6)	0.650

Data are mean ± standard deviation unless otherwise stated.

^†^Number of drugs except for anti-PD drugs.

^‡^During 3 months before the index date.

ATC, Anatomical Therapeutic Chemical; NA, not applicable; PD, Parkinson's disease.

### 3.2. Patient characteristics

Among All patients, the mean ages for the ≥75-year and < 75-year groups were 81.3 and 60.9 years, respectively. There were similar proportions of men and women among patients < 75 years, but more women than men among elderly patients ≥75 years (Table 1A). Elderly patients ≥75 years had a higher rate of comorbidities (such as constipation, pain, and cognitive disorder) and were prescribed fewer anti-PD drug types compared with patients < 75 years. Compared with elderly patients, a numerically higher ratio of patients < 75 years was prescribed < 100 mg levodopa as the maximum dose during the observation period (Supplementary Figure 2). The proportions of newly initiated PD treatment patients with comorbidities such as constipation, insomnia, pain, and cognitive disorder tended to be high, particularly among elderly patients ≥75 years (Table 1B). The proportions of patients receiving concomitant non–anti-PD drugs by comorbidities are shown in Supplementary Table 4.

### 3.3. Anti-PD drug prescribing patterns: all patients

**Figure 2:**
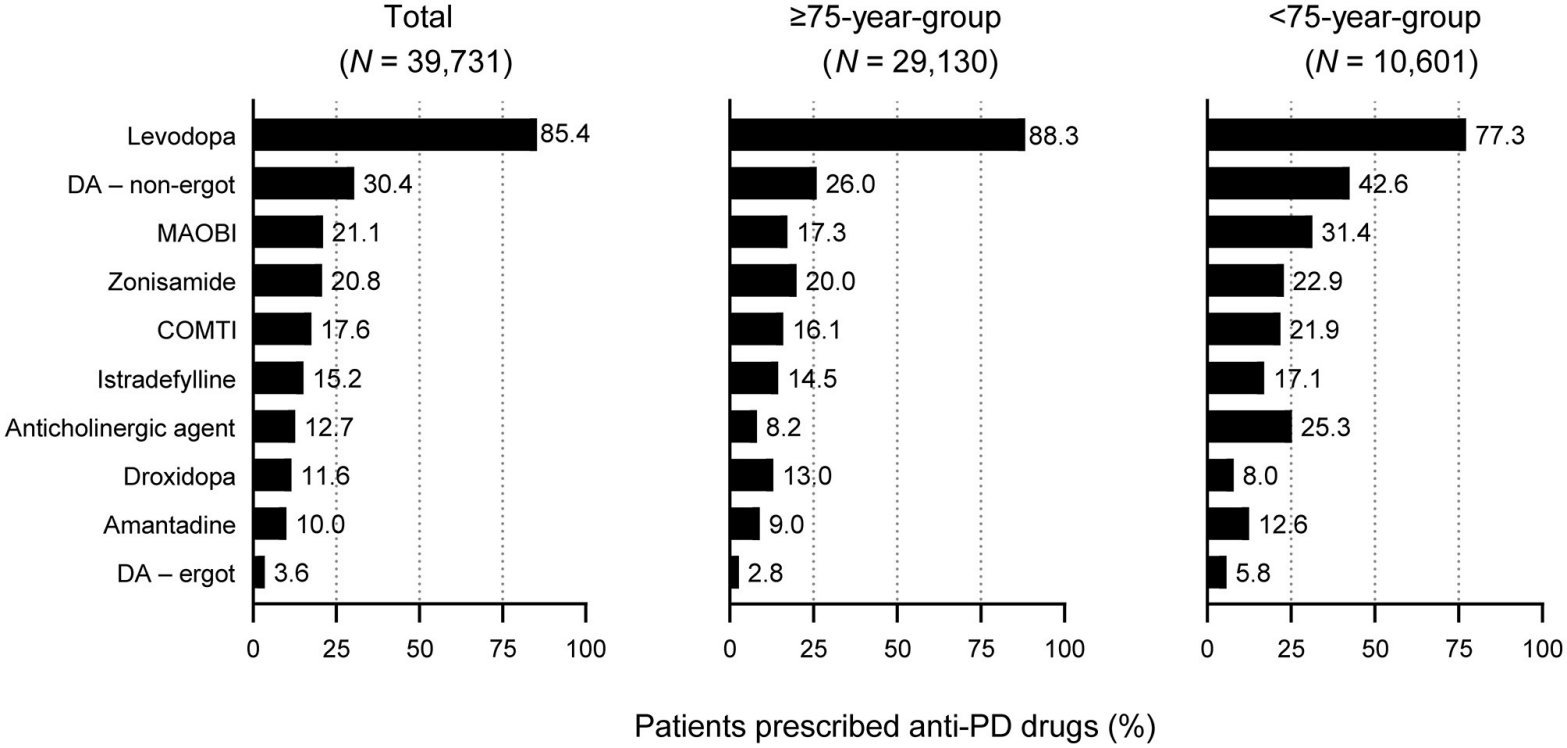
Prescription of anti-PD drug types in patients with PD included in this analysis: All patients by age group. For all drugs, differences between the ≥75-year group and the < 75-year group were statistically significant (*p* < 0.05). COMTI, catechol-*O*-methyltransferase inhibitor; DA, dopamine agonist; MAOBI, monoamine oxidase type B inhibitor; PD, Parkinson's disease.

All patients were most commonly prescribed levodopa during the observation period. A higher proportion of elderly patients ≥75 years were prescribed levodopa than patients < 75 years (88.3% vs. 77.3%, respectively, *p* < 0.05) (Figure 2). In contrast, fewer elderly patients ≥75 years were prescribed anti-PD drugs such as non-ergot DAs, MAO-B inhibitors, and anticholinergic agents than those < 75 years (Figure 2), and elderly patients ≥75 years were prescribed a smaller number of anti-PD drug types compared with younger patients (2.2% vs. 2.6%, respectively, *p* < 0.05) (Table 1A).

**Figure 3:**
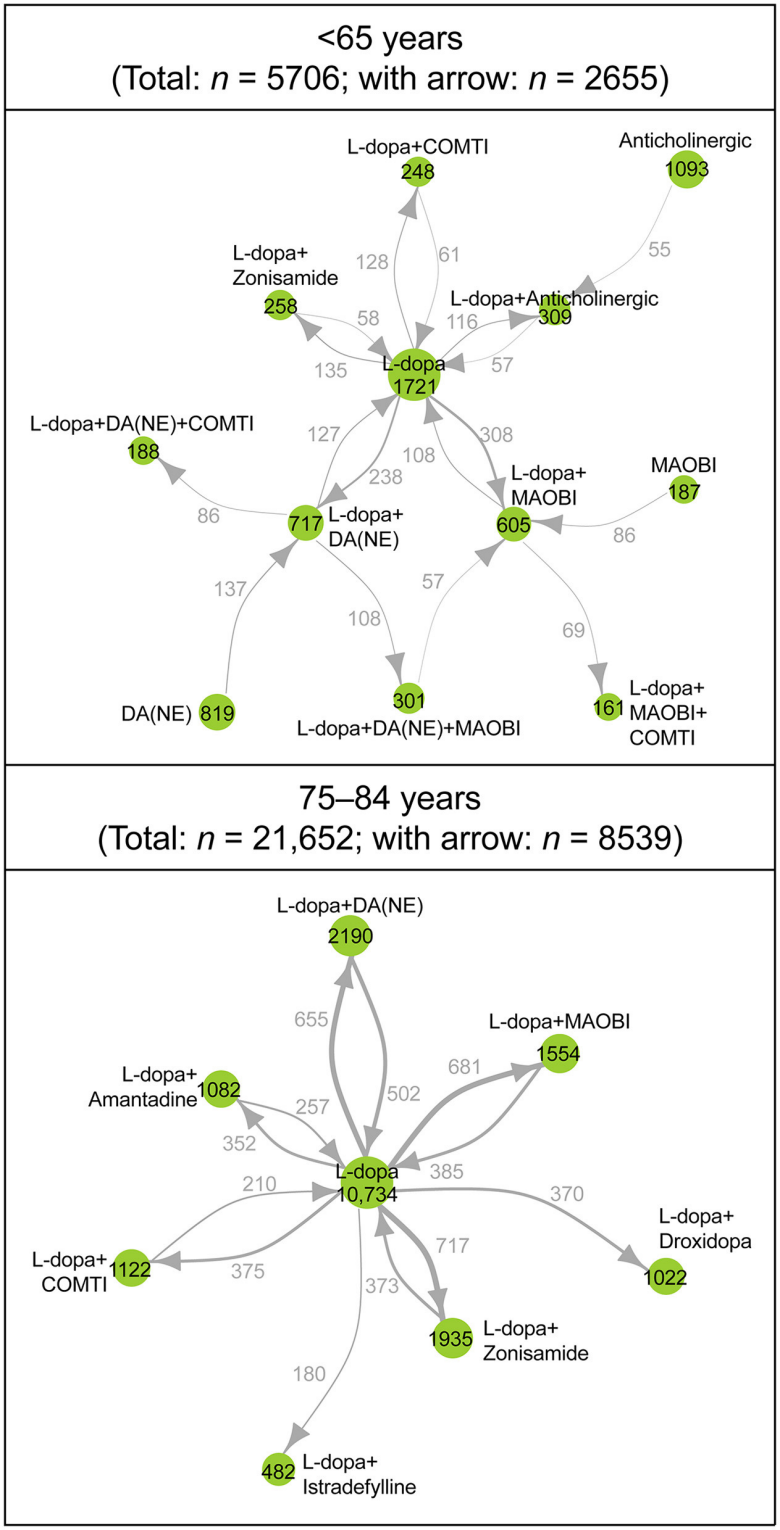
Longitudinal analysis of the type and combinations of anti-PD drugs prescribed during the observation period: patients < 65 years (*n* = 5,706) and patients 75–84 years (*n* = 21,652). Each node represents a prescribing pattern, and the size of each node represents the number of patients with each prescribing pattern. The thickness of each arrow represents the number of patients who transitioned between prescribing patterns. Arrows are only shown for those that represent ≥2% of patients in each age group. The number of nodes and arrows in patients < 65 years (12 and 17 arrows, respectively), 75–84 years (8 and 12, respectively). COMTI, catechol-*O*-methyltransferase inhibitor; DA, dopamine agonist; L-dopa, levodopa; MAOBI, monoamine oxidase type B inhibitor; NE, non-ergot; PD, Parkinson's disease.

In the network analysis of prescribing patterns, most patients switched from levodopa monotherapy to an adjunct prescription pattern that included a non-ergot DA, MAO-B inhibitor, and zonisamide (Figure 3, Supplementary Figure 3). Prescribing patterns in the ≥75-year group were levodopa centered and less complex compared with those < 75 years. Patients 65–74 years had the most complex prescribing patterns (Figure 3, Supplementary Figure 3). In the network diagram, there were fewer nodes and arrows in patients 75–84 years (8 and 12, respectively) and ≥85 years (8 and 13, respectively) compared with patients < 65 years (12 and 17, respectively) and 65–74 years (13 and 18, respectively). In addition, patients < 65 years were prescribed three-drug combinations or levodopa with COMT inhibitors or an anticholinergic agent as well as monotherapy with non-ergot DAs or MAO-B inhibitors. Patients 65–74 years had the most complex prescribing patterns (Figure 3, Supplementary Figure 3). The prescription of anti-PD drugs by comorbidities is shown in Supplementary Table 5.

### 3.4. Anti-PD drug prescribing patterns: patients who newly initiated PD treatment

**Figure 4:**
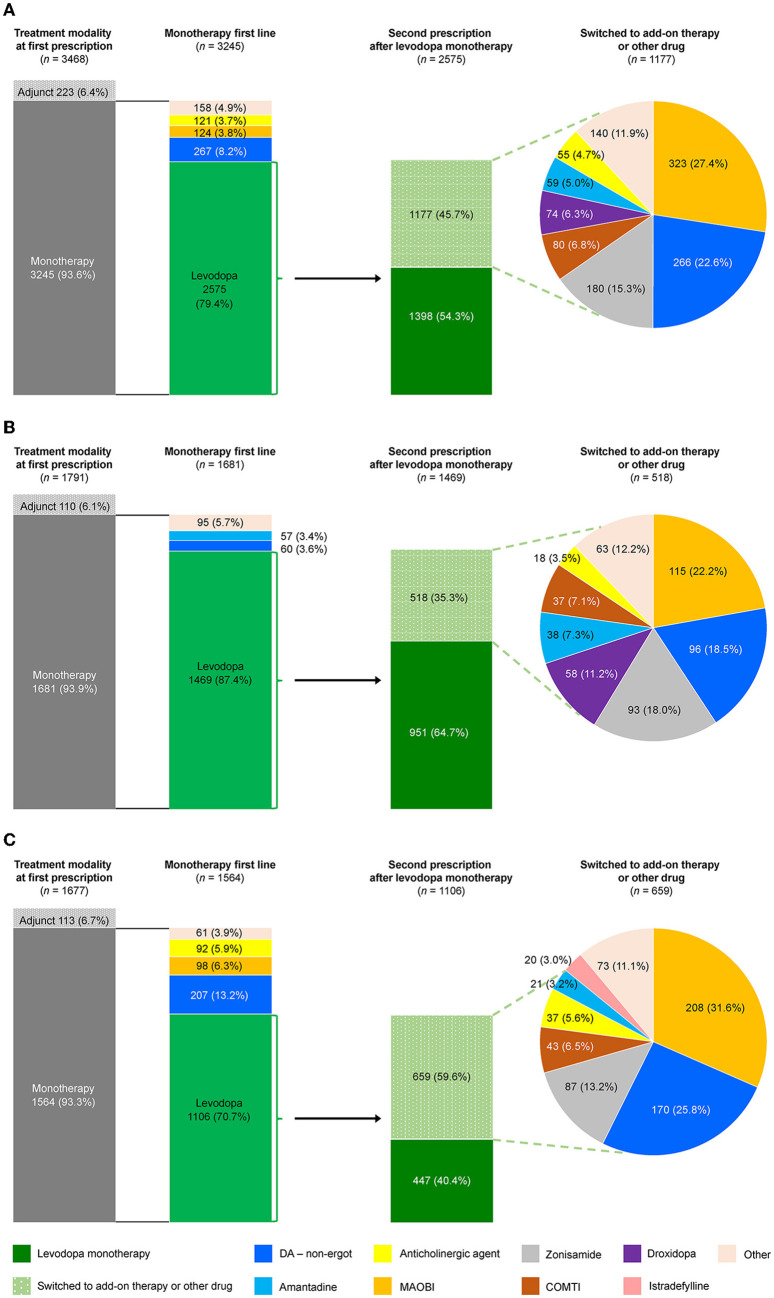
Prescribing patterns for anti-PD drugs in patients who newly initiated PD treatment by age group. **(A)** Total, **(B)** ≥75-year group, and **(C)** < 75-year group. COMTI, catechol-*O*-methyltransferase inhibitor; DA, dopamine agonist; MAOBI, monoamine oxidase type B inhibitor; PD, Parkinson's disease.

**Figure 5:**
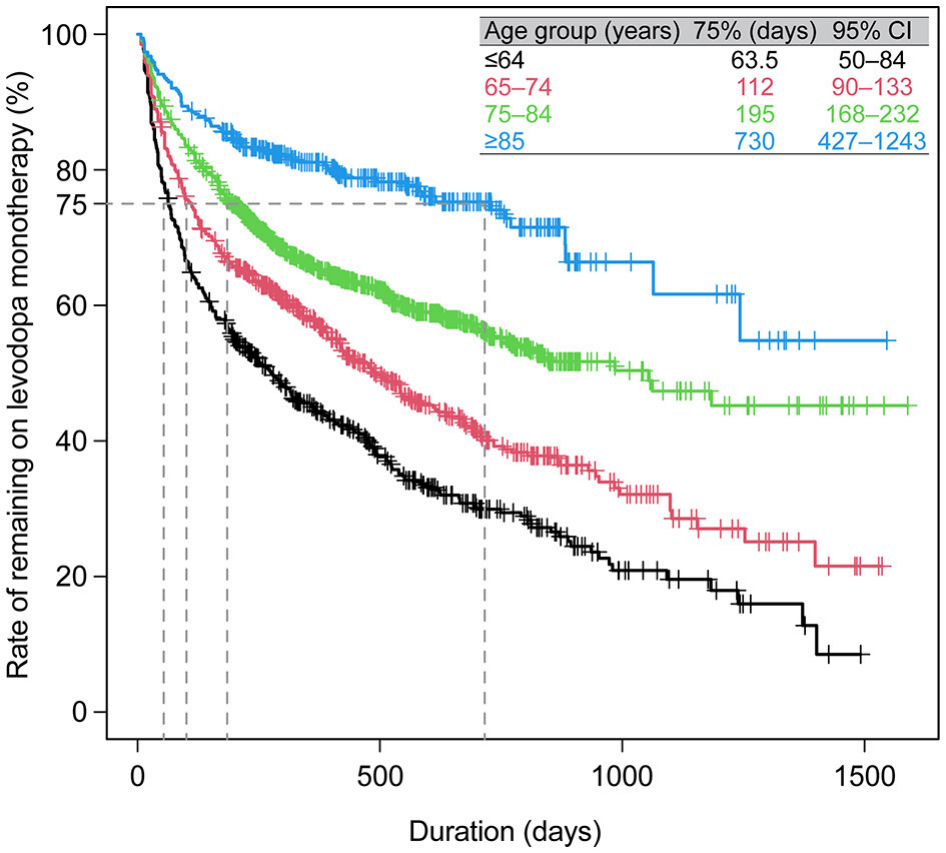
Duration of levodopa monotherapy in patients who newly initiated PD treatment with a first prescription for levodopa monotherapy (*N* = 2,575). CI, confidence interval; PD, Parkinson's disease.

Prescribing patterns were also analyzed for the subgroup of All patients who newly initiated PD treatment during the observation period. Of the 3,468 patients who newly initiated PD treatment, 93.6% (3,245) started on monotherapy, and of those, 79.4% (2,575) started on levodopa (Figure 4A). Of the patients prescribed levodopa monotherapy, 54.3% (1,398) stayed on monotherapy during the observation period, and 45.7% (1,177) transitioned to levodopa adjunct therapy. Patients who stayed on levodopa monotherapy were slightly older and had higher rates of constipation (45.5% vs. 36.4%, *p* < 0.05), insomnia (26.0% vs. 19.6%, *p* < 0.05), and cognitive disorder (12.8% vs. 4.7%, *p* < 0.05) than those who switched to adjunct therapy, respectively (Supplementary Table 6). In terms of prescribing patterns by age, a higher proportion of elderly patients ≥75 years initiated and stayed on levodopa monotherapy vs. those < 75 years (Figures 4B, C, respectively). Moreover, older patients stayed on levodopa monotherapy longer than younger patients (Figure 5, Supplementary Table 7). The duration of continuation for 75% of patients who newly initiated PD treatment with a first prescription for non-ergot DA monotherapy was 102.5–147 days (Supplementary Figure 4). The most frequently used adjunct therapies to levodopa were MAO-B inhibitors, non-ergot DAs, and zonisamide (Figure 4A). These findings were consistent in both age groups, except that more patients in the ≥75-year group had levodopa monotherapy as a first prescription and slightly fewer elderly patients ≥75 years were prescribed MAO-B inhibitors and non-ergot DAs than younger patients < 75 years (Figure 4). In addition, compared with younger patients < 75 years, elderly patients ≥75 years were prescribed a wider variety of other anti-PD drugs, including droxidopa and amantadine.

The factors significantly associated with a levodopa monotherapy prescription in patients who newly initiated PD treatment were older age and cognitive impairment (Table 2). The factors significantly associated with the longer duration of levodopa monotherapy were older age and cognitive disorder, and the factors associated with the shorter duration of levodopa monotherapy were female sex and hallucination (Supplementary Table 7). However, it should be noted that the number of patients with hallucinations was small (levodopa monotherapy, *n* = 7; levodopa adjunct therapy, *n* = 8). In addition, although the number of patients was small, non-ergot DA monotherapy was less likely to be selected as the first prescription pattern in elderly patients (Supplementary Table 8). There was no significant effect of age or comorbidities on the duration of non-ergot DA monotherapy (Supplementary Table 9, Supplementary Figure 4). Demographics and clinical characteristics of elderly patients ≥75 years receiving DA adjunct therapy after levodopa monotherapy are shown in Supplementary Table 10.

**Table 2:** Factors associated with a prescription for levodopa monotherapy in patients who newly initiated PD treatment (*N* = 3,467).

**Variable**	**Odds ratios**	**95% CI**	***p*-value**
Age (years)	1.057	1.040–1.075	0.000
Sex (ref. female)	0.850	0.718–1.007	0.060
Comorbidities^†^, middle category (ref. no comorbidity)			
Depression and anxiety	1.028	0.806–1.314	0.822
Hallucination	0.420	0.139–1.241	0.114
Cognitive disorder	2.088	1.495–2.961	0.000
Number of concomitant drugs^†^	0.994	0.971–1.017	0.616
Charlson Comorbidity Index^†^	1.017	0.962–1.076	0.558
Database (ref. JMDC database)			
Elderly	0.917	0.621–1.351	0.661
NHI	0.877	0.664–1.156	0.352

^†^During 3 months before the index date.

CI, confidence interval; NHI, National Health Insurance; PD, Parkinson's disease; ref, reference.

### 3.5. Anti-PD drug prescribing patterns: patients who increased their levodopa dose to ≥300 mg

**Figure 6:**
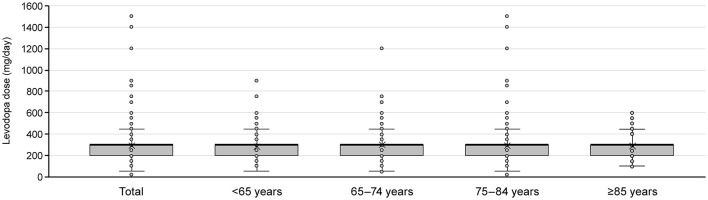
Levodopa dose at the time of switching to adjunct therapy for patients who newly initiated PD treatment with levodopa monotherapy by age group. Box plots show mean, median, quartiles, minimum, maximum, and outliers. PD, Parkinson's disease.

Of the patients who initiated levodopa monotherapy and transitioned to levodopa adjunct therapy, most were prescribed levodopa 300 mg/day at the time the second anti-PD drug was added, regardless of age (Figure 6). Therefore, we focused on those patients who added a second prescription after their daily levodopa dose increased to ≥300 mg. Among All patients, 8,677 patients increased their levodopa dose to ≥300 mg/day during the observation period (Supplementary Figure 5A). The characteristics of patients who increased their levodopa dose to ≥300 mg are shown in Supplementary Table 11.

After increasing their levodopa dose to ≥300 mg, 66.9% (3,569) of All patients who were on levodopa monotherapy remained on levodopa monotherapy during the observation period. Elderly patients ≥75 years (74.2% [2,870]) were more likely to continue levodopa monotherapy than those < 75 years (47.7% [699]) (Supplementary Figures 5B, C). The most commonly prescribed drugs when patients transitioned from levodopa monotherapy to adjunct therapy with levodopa ≥300 mg were MAO-B inhibitors, non-ergot DAs, and zonisamide (Supplementary Figure 5A). In elderly patients ≥75 years, zonisamide followed by MAO-B inhibitors and non-ergot DAs were the most commonly prescribed adjunct therapies, although the proportion of elderly patients who transitioned to adjunct therapy was low overall (Supplementary Figures 5B, C). Droxidopa and amantadine were also prescribed to a certain extent, and there was a tendency for many types of anti-PD drugs to be prescribed without substantial bias.

## 4. Discussion

This analysis of real-world prescribing patterns of anti-PD drugs in Japan is the first to include nationwide medical claims, which used the Elderly database to focus on elderly patients (≥75 years). Unlike previous studies in Japan, which examined insurance claims from company employees and a hospital administrative database (12–14), this study was able to calculate the prevalence of PD among elderly patients and analyze anti-PD drug prescribing patterns longitudinally. As Japan is a country with a rapidly aging society, findings from this study may provide insights into PD treatment for elderly patients in other countries. In alignment with global estimates (1) and previous Japanese studies (18, 19), the prevalence of PD in this study was highest in patients ≥75 years (1.21/100 people from June 2020 to May 2021). Age is a risk factor for several non-motor symptoms of PD such as constipation, cognitive disorder, hallucinations, or insomnia (20–23). In this study, there was a high proportion of elderly patients with these symptoms, which suggests that they were receiving treatment for these conditions.

Consistent with previous studies in Japan [which were conducted in 2005–2010 (12) and 2008–2016] (13, 14), studies in other countries (9, 24, 25), and treatment guidelines (11, 26, 27), levodopa was the most commonly prescribed anti-PD drug in this study, with non-ergot DAs, MAO-B inhibitors, and zonisamide added or subtracted to levodopa monotherapy as needed. Patients < 75 years, especially those aged 65–74 years, had the most heterogeneous prescribing patterns, starting with non-levodopa monotherapy and eventually transitioning to three-drug combinations. In contrast, elderly patients ≥75 years had simpler prescribing patterns that were centered predominantly on levodopa. Elderly patients experience more severe PD symptoms from the time of diagnosis than younger patients, motor symptoms progress faster, and they have a higher risk of developing hallucinations and dementia (28, 29). Hence, the focus on levodopa for elderly patients is most likely because levodopa is more effective at improving motor symptoms (11, 26, 27) and because levodopa has relatively fewer side effects than other drugs, which is important for elderly patients who have a high risk of cognitive disorder and psychiatric symptoms (7, 11, 30). Consistent with this, the factors significantly associated with a levodopa prescription and continued use of levodopa in this study were older age and the presence of cognitive disorder. Additionally, our results suggested that prescribing patterns may be simpler in the elderly because of the need to avoid polypharmacy and because of the concerns about drug side effects associated with decreasing metabolic function (6, 7).

In this study, we also analyzed prescribing patterns for patients who newly initiated PD treatment during the observation period in order to evaluate initial PD treatment in elderly patients; 79.4% of those who newly initiated PD treatment were prescribed levodopa during the observation period. Similar to this study, an analysis of prescribing patterns in newly diagnosed patients from the United States showed that levodopa monotherapy was the most common treatment, 70% of patients on monotherapy were prescribed levodopa, and 58% stayed on levodopa monotherapy (25). We have extended these findings and shown that, in addition, elderly patients were more likely to initiate and stay on levodopa monotherapy compared with younger patients. In both age groups, the most common drugs added as a second prescription after levodopa monotherapy were MAO-B inhibitors, non-ergot DAs, and zonisamide. MAO-B inhibitors and non-ergot DAs have been reported to improve motor symptoms as well as non-motor symptoms such as mood disorder, pain, sleep disorders, and quality of life (31–35). In addition, zonisamide has been approved for the treatment of PD in Japan and is expected to improve tremors in addition to improving general motor symptoms (36–38). Anticholinergic agents are also prescribed to treat tremors but are avoided in those ≥75 years, most likely because of the potential risk of decline in memory and cognitive function, and falls (11). Compared with younger patients, more elderly patients in this study were also prescribed droxidopa, which may be used as a treatment for neurogenic orthostatic hypotension (39) and freezing of gait (40). Thus, our findings suggest that although levodopa monotherapy is the most commonly used drug, there are many treatment options in Japan, and that combination therapy that utilizes the characteristics of each drug may be used depending on individual patients' needs, especially in elderly patients.

In addition to evaluating prescribing patterns, we also analyzed the levodopa dose in patients who switched from levodopa monotherapy to adjunct therapy. Although Japanese treatment guidelines also recommend levodopa-centered therapy, there is no clear statement on the timing or dosage of levodopa with adjunct therapy after initiating treatment with levodopa monotherapy. We expected that elderly patients with a relatively low risk of developing motor complications would receive levodopa monotherapy at higher doses compared with younger patients. However, regardless of age, the second prescription after levodopa monotherapy occurred when the levodopa dose had increased to ≥300 mg in this study. While the reasons for this are unclear, we believe that because motor complications can also occur in elderly patients (41), it is likely that patients of any age add on other anti-PD drugs to levodopa at a dose of 300 mg to avoid motor complications with higher levodopa doses (42). The adjunct therapies with zonisamide and droxidopa that were observed in elderly patients indicate that physicians aim to manage patients' symptoms of dopamine resistance, such as frozen gait (40) and tremors (36–38), as well as the adverse effects of dopaminergic agents such as orthostatic hypotension and hallucinations/delusions, that can occur more frequently in patients of advanced age.

The limitations of this analysis are that because the data are based on medical claims, it is not possible to confirm a patient's actual use of a prescribed drug and there is no information on PD severity or duration, which may affect the choice of drugs prescribed. Thus, the analysis of drug preference by PD severity and comorbidities could not be adequately assessed in this study. In addition, because data were combined from three nationwide claims databases, the analysis population is not an exact reflection of the age distribution of patients with PD in Japan. However, by including the Elderly database, our study has been able to provide meaningful information on a clinically relevant population for PD. Finally, because the observation period for the databases was short (3 or 4 years), it was not possible to evaluate long-term prescription patterns and characteristics. In order to conduct a long-term analysis, a national database that covers the entire Japanese population is needed.

## 5. Conclusion

In Japan, which has one of the world's most prominent aging populations, this is the first large-scale database study to include an elderly population ≥75 years for the evaluation of PD prescribing patterns and treatment. Compared with patients < 75 years, elderly patients were more likely to be prescribed levodopa, to stay on levodopa for longer, and had simpler prescribing patterns. The factors significantly associated with levodopa monotherapy and continued use of levodopa were older age and the presence of cognitive disorder. Elderly patients initiated PD treatment with levodopa monotherapy, and a second therapy such as MAO-B inhibitors, non-ergot DAs, or zonisamide was added to levodopa when the levodopa dose increased to 300 mg, similar to younger patients. In addition, adjunct therapies were diverse, suggesting that a variety of drugs can be prescribed for PD in Japan, and that tailor-made treatment is implemented according to individual patient characteristics.

## Data availability statement

The data that support the findings of this study are available from DeSC Healthcare, Inc. and JMDC., Ltd. but were used under license for the current study; therefore, restrictions apply and the data are not publicly available. For inquiries about access to the data set used in this study, please contact DeSC Healthcare, Inc. (https://desc-hc.co.jp/en) and JMDC Inc. (https://www.jmdc.co.jp/en/).

## Ethics statement

The studies involving human participants were reviewed and approved by the Research Institute of Healthcare Data Science Institutional Review Board (No. RI2021024) in Japan. Written informed consent for participation was not required for this study in accordance with the national legislation and the institutional requirements.

## Author contributions

MS, YK, and AH were involved in conceptualization, formal analysis, investigation and methodology, and data visualization. AH was also involved in resources and software for the study. MA was involved in funding acquisition, investigation, project administration, and supervision. SF was involved in project administration and supervision. All authors participated in the interpretation of study results and in the drafting, critical revision, and approval of the final version of the manuscript.
