# Supplementary material for: Prescribing patterns and determinants for elderly patients with Parkinson's disease in Japan: a retrospective observational study using insurance claims databases
Source: Front Neurol. 2023 Jun 23;14:1162016. doi: 10.3389/fneur.2023.1162016 (PMC10327598; doi:10.3389/fneur.2023.1162016)
Supplement: Supplementary file 1 [file Data_Sheet_1.docx]

Supplementary material

**SUPPLEMENTARY Table 1** Classification of drugs prescribed for Parkinson’s disease.

**SUPPLEMENTARY Table 2** Comorbidities included in this analysis.

**SUPPLEMENTARY Table 3** Prevalence of Parkinson’s disease by age group: All patients.

**SUPPLEMENTARY Table 4** Concomitant drug prescribing by ATC main category: All patients.

**Supplementary Table 5** Prescription of anti-PD drug by comorbidities: All patients.

**SUPPLEMENTARY Table 6** Demographics and clinical characteristics of patients who newly initiated PD treatment and who stayed on levodopa monotherapy during the observation period or switched to adjunct therapy.

**SUPPLEMENTARY Table 7** Factors associated with duration of levodopa monotherapy in patients who newly initiated PD treatment (*N* = 2,575).

**SUPPLEMENTARY Table 8** Factors associated with a prescription for non-ergot DA monotherapy in patients who newly initiated PD treatment (*N* = 3,467).

**SUPPLEMENTARY Table 9** Factors associated with duration of non-ergot DA monotherapy in patients who newly initiated PD treatment (*N* = 267).

**SUPPLEMENTARY TABLE 10** Demographics and clinical characteristics of elderly patients ≥75 years who newly initiated PD treatment and who stayed on levodopa monotherapy during the observation period or switched to levodopa and non-ergot DA adjunct therapy.

**SUPPLEMENTARY Table 11** Demographics and clinical characteristics of patients who increased levodopa dose from <300 mg to ≥300 mg during the observation period.

**SUPPLEMENTARY FIGURE 1** Flow diagram.

**SUPPLEMENTARY FIGURE 2** Maximum levodopa doses prescribed by age group during the observation period (All patients and those who newly initiated PD treatment).

**SUPPLEMENTARY FIGURE 3** Longitudinal analysis of the type and combinations of anti-PD drugs prescribed during the observation period: Patients Total (*N* = 39,731), patients 65–74 years (*N* = 5,312), and patients ≥85 years (*N* = 8,162).

**supplementary Figure 4** Duration of non-ergot DA monotherapy in patients who newly initiated PD treatment with a first prescription for non-ergot DA monotherapy (*N* = 267).

**SUPPLEMENTARY Figure 5** Treatment modalities and drug prescriptions when patients who increased their levodopa dose to ≥300 mg. (**A**) Total, (**B**) ≥75-year-group, and (**C**) <75-year-group.

**SUPPLEMENTARY Table 1** Classification of drugs prescribed for Parkinson’s disease.

| **Drug** | **Receipt code from computerized processing system** | **ATC code** |
| --- | --- | --- |
| Levodopa  Levodopa  Levodopa carbidopa hydrate  Levodopa benserazide hydrochloride  Levodopa carbidopa hydrate entacapone | 620006042, 620006043, 620008660, 620008661, 620008662, 620008889, 620008890 | N04BA01 |
|  | 620123601, 620123701, 620123801, 620123901, 620124001, 620124101, 620124201, 620124301, 620124401, 620124501, 620124601, 621865201, 622050801, 622051301, 622067101, 622080201, 622509401, 622711600, 622711800 | N04BA02 |
|  | 620008030, 620008659, 622325500, 622711700 | N04BA02 |
|  | 622375701, 622375801 | N04BA03 |
| Dopamine agonist – ergot  Bromocriptine mesilate  Pergolide mesilate  Cabergoline | 610422106, 620005063, 620005096, 620006069, 620006743, 620008579, 620008688, 620009237, 620120902, 620121001, 620121101, 620121102, 620121401, 620121701, 620121802, 620121902, 620121904, 620122001 | N04BC01 |
|  | 620121801 | NA |
|  | 610406392, 610406393, 620002124, 620002125, 620002823, 620002857, 620002858, 620004067, 620004068, 621642002, 621642103, 621690101 | N04BC02 |
|  | 620006684, 620006685, 622711100, 622711200 | NA |
|  | 610432023, 610432024, 620005369, 620005370, 620005371, 620005372, 620005375, 620005376, 620005377, 620005378, 620005379, 620008002, 622711400, 622711500 | N04BC06 |
|  | 620005373, 620006667, 620006668, 620008001 | NA |
| Dopamine agonist – non-ergot  Talipexole hydrochloride  Ropinirole  Pramipexole hydrochloride hydrate  Apomorphine hydrochloride hydrate  Rotigotine | 610409337 | N04BC |
|  | 620004415, 620004416, 620004417, 622183601, 622183701, 622492301, 622492401, 622492501, 622500001, 622500101, 622500201, 622582201, 622582301, 622583401, 622583501, 622656201, 622656301, 622687201, 622687301, 622687401, 622687501, 622687601 | N04BC04 |
|  | 620000435, 620000436, 622069601, 622069701, 622226701, 622226801, 622228701, 622228801, 622229801  622229901, 622231601, 622231701, 622233901, 622234001, 622235901, 622236001, 622238001, 622238101, 622239601, 622239701, 622245201, 622245301, 622247301, 622247401, 622249201, 622249301, 622253101, 622253201, 622253501, 622253601, 622254501, 622254601, 622256401, 622256501, 622257401, 622257501, 622272601, 622272701, 622511201, 622511301, 622513801, 622513901, 622520201, 622520301, 622524801, 622524901, 622528201, 622528301, 622530001, 622530101, 622691900 | N04BC05 |
|  | 622263001, 622263101, 622263201, 622263301, 622692000 | NA |
|  | 622149301 | N04BC07 |
|  | 622163501, 622163601, 622163701, 622163801, 622478301 | N04BC09 |
| Monoamine oxidase type B inhibitor  Selegiline hydrochloride  Rasagiline mesilate  Safinamide mesilate | 610421338, 620003975, 620005364, 620005511, 620008494, 622711300 | N04BD01 |
|  | 622624401, 622624501 | N04BD02 |
|  | 622696101 | N04BD03 |
| Catechol-*O*-methyltransferase inhibitor  Entacapone  Opicapone | 620004853, 622478401, 622490101, 622498501, 622503001 | N04BX02 |
|  | 622845500, 622878901 | NA |
|  | 622821701 | N04BX04 |
| Anticholinergic agent  Trihexyphenidyl hydrochloride  Biperiden hydrochloride  Biperiden lactate  Profenamine hydrochloride  Profenamine hibenzate | 610454002, 611240304, 611240415, 611240423, 611240425, 620003078, 620004382, 620004384, 620004548, 620006589, 620008311, 620008663, 620119903, 620119906, 620119907, 620119919, 620119923, 620119927, 620120303 | N04AA01 |
|  | 620004556, 620005847, 622319200 | NA |
|  | 610444006, 610444007, 620000066, 620000067, 620000243, 620004893, 620004894, 620005147, 620117802, 620118102, 620118203, 620118402, 621218501, 621392101 | N04AA02 |
|  | 620006075 | NA |
|  | 611240172, 611240173 | N04AA05 |
|  | 610463153 |  |
| Zonisamide | 611130087, 620001972, 620009098, 621390801, 621390901, 622400401, 622589801, 622673201, 622709200, 622709300 | N03AX15 |
| Istradefylline | 622245801 | N04CX01 |
| Amantadine | 610461044, 610461047, 611240079, 611240080, 620002335, 620004505, 620008286, 620116802, 620116809, 620117101, 620117103, 620117110, 620117112, 620117114, 620117401, 620117402, 620117404, 620117412, 620117413, 620117414, 622710800 | N04BB01 |
|  | 610461046, 611240122, 611240123, 620003077, 620003267, 620003268, 620005903, 620006587, 620008280, 620116701, 620117107, 620117410, 620116702, 622309500, 620116805 | NA |
| Droxidopa | 620004007, 620004008, 620004009, 620004010, 620005044, 620005045, 620005046, 620006676, 620006677, 620006678, 622061101, 622061201 | C01CA27 |
|  | 622710900, 622711000 |  |

ATC, Anatomical Therapeutic Chemical; NA, not applicable.

**SUPPLEMENTARY Table 2** Comorbidities included in this analysis.

| **Comorbidity** | **Disease code** |
| --- | --- |
|  |  |
| Constipation | 8850626, 8847697, 8833027, 8834176, 5640018, 5640016, 8847705, 8837133, 5640007, 8837785, 5640011, 8840042, 8849551, 8849481 |
| Insomnia | 8839792 |
| Pain | 8838060, 8846220, 8849544, 7213016, 8845840, 7153018, 8850089 |
| Cognitive disorder | 8831737, 7809004, 7809005, 7809006, 7809007, 7809026, 8833248, 7809021, 8842548, 8842549, 8842550, 8842551, 3310002, 8842308, 8849974, 8847912, 8847913, 8842565, 8842571, 8842608, 8842625, 8842626, 8845840, 8842575, 2901004, 8842591, 2902001, 8842615, 8842618, 2902006, 8842637, 8842638, 8842639, 2902008, 2902012 |
| Depression and anxiety | 3004001, 2961003, 2961005, 8832914, 8850416, 2961017, 2961020, 8835867, 8835868, 2961023, 8837202, 8837626, 2980001, 2961027, 8831486, 8832918, 2961024, 8837627, 2961025, 8839033, 2961026, 8839040, 8839041, 8832574, 8833458, 8843724, 8834492, 8842740, 8845669, 8836029, 3002024, 8836934, 8837086, 8837121, 3002018, 8839428, 8839939, 8833894, 8836575, 8836634, 3000003, 8844095, 8844096, 3004024, 3000004, 8839596, 3000010, 8839600 |
| Hallucination | 2989002, 7801001, 2971002, 2971003, 2979001, 3009003, 8833547, 2971005, 8834119, 2971007, 2971009, 3002009, 3002010, 8835222, 2972002, 2971001, 2971012, 8840579, 8840580, 7800001 |

**SUPPLEMENTARY Table 3** Prevalence of Parkinson’s disease by age group: All patients.

|  | **≥75 years** | **<75 years** | |
| --- | --- | --- | --- |
|  | **Elderly database**  ***N* = 29,130** | **NHI database (self-employed/unemployed)**  ***N* = 5,364** | **JMDC database (company/government employees and their dependents)**  ***N* = 5,237** |
| Prevalence  (June 2016 to May 2021), (%) | 1.14 | 0.31 | 0.07 |
| Prevalence  (June 2020 to May 2021), (%) | 1.21 | 0.37 | 0.07 |
| ≤54 years | NA | 0.10 | 0.03 |
| 55–64 years | NA | 0.30 | 0.13 |
| 65–74 years | NA | 0.58 | 0.32 |
| 75–84 years | 1.13 | NA | NA |
| ≥85 years | 1.36 | NA | NA |

NA, not applicable; NHI, National Health Insurance.

**SUPPLEMENTARY Table** **4** Concomitant drug prescribing by ATC main category: All patients.

| **ATC main category** | **Total**  ***N* = 39,731** | **≥75 years**  ***N* = 29,130** | **<75 years**  ***N* = 10,601** | **Group Difference**  ***P*-value** |
| --- | --- | --- | --- | --- |
| Alimentary tract and metabolism | 36,887 (92.8) | 27,567 (94.6) | 9,320 (87.9) | 0.000 |
| Blood and blood forming organs | 19,947 (50.2) | 15,529 (53.3) | 4,418 (41.7) | 0.000 |
| Cardiovascular system | 28,668 (72.2) | 22,496 (77.2) | 6,172 (58.2) | 0.000 |
| Dermatologicals | 3,025 (7.6) | 2,419 (8.3) | 606 (5.7) | 0.000 |
| Genito urinary system and sex hormones | 11,716 (29.5) | 9,205 (31.6) | 2,511 (23.7) | 0.000 |
| Systemic hormonal preparations, excluding sex hormones and insulins | 4,013 (10.1) | 2,985 (10.2) | 1,028 (9.7) | 0.112 |
| Antiinfectives for systemic use | 13,778 (34.7) | 10,283 (35.3) | 3,495 (33.0) | 0.000 |
| Antineoplastic and immunomodulating agents | 1,322 (3.3) | 952 (3.3) | 370 (3.5) | 0.289 |
| Musculo-skeletal system | 16,119 (40.6) | 10,966 (37.6) | 5,153 (48.6) | 0.000 |
| Nervous system | 31,295 (78.8) | 23,402 (80.3) | 7,893 (74.5) | 0.000 |
| Antiparasitic products, insecticides, and repellents | 1,274 (3.2) | 946 (3.2) | 328 (3.1) | 0.462 |
| Respiratory system | 19,558 (49.2) | 14,207 (48.8) | 5,351 (50.5) | 0.003 |
| Sensory organs | 2,230 (5.6) | 1,872 (6.4) | 358 (3.4) | 0.000 |
| Various | 3,222 (8.1) | 810 (2.8) | 2,412 (22.8) | 0.000 |

Data are n (%).

ATC, Anatomical Therapeutic Chemical.

**Supplementary Table 5** Prescription of anti-PD drug by comorbidities: All patients.

| Characteristic | **Constipation** | **Insomnia** | **Pain** | **Cognitive Disorders** | **Depression and anxiety** | **Hallucination** | **Orthostatic hypotension** |
| --- | --- | --- | --- | --- | --- | --- | --- |
|  | ***N = 32,909*** | ***N = 21,607*** | ***N = 20,777*** | ***N = 15,871*** | ***N = 14,195*** | ***N = 2,114*** | ***N = 2,659*** |
| Male, *n* (%) | 11,779 (35.8) | 7,331 (33.9) | 6,776 (32.6) | 5,953 (37.5) | 4,191 (29.5) | 762 (36.0) | 1,276 (48.0) |
| Age (years), mean ± SD (range) | 77.2 ± 9.9  (30–104) | 76.4 ± 11.1  (30–102) | 77.7 ± 9.1  (30–102) | 80.4 ± 7.2  (34–102) | 75.2 ± 12.0  (30–102) | 78.4 ± 9.4  (30–101) | 76.6±9.9  (30–97) |
| Age, *n* (%) |  |  |  |  |  |  |  |
| ≤54 | 1,206 (3.7) | 1,272 (5.9) | 557 (2.7) | 91 (0.6) | 1,100 (7.7) | 71 (3.4) | 113 (4.2) |
| 55–64 | 2,199 (6.7) | 1,509 (7.0) | 1,224 (5.9) | 415 (2.6) | 1,185 (8.3) | 84 (4.0) | 190 (7.1) |
| 65–74 | 3,792 (11.5) | 2,335 (10.8) | 2,293 (11.0) | 1,102 (6.9) | 1,660 (11.7) | 200 (9.5) | 290 (10.9) |
| 75–84 | 18,921 (57.5) | 12,217 (56.5) | 12,531 (60.3) | 9,815 (61.8) | 7,677 (54.1) | 1,261 (59.6) | 1,596 (60.0) |
| ≥85 | 6,791 (20.6) | 4,274 (19.8) | 4,172 (20.1) | 4,448 (28.0) | 2,573 (18.1) | 498 (23.6) | 470 (17.7) |
| Prescribing patten, *n* (%) |  |  |  |  |  |  |  |
| Levodopa | 16,163 (49.1) | 10,322 (47.8) | 10,596 (51.0) | 8,681 (54.7) | 6,899 (48.6) | 1,091 (51.6) | 1,000 (37.6) |
| DA – non-ergot | 1,905 (5.8) | 1,743 (8.1) | 1,517 (7.3) | 689 (4.3) | 1,222 (8.6%) | 99 (4.7) | 100 (3.8) |
| MAOBI | 404 (1.2) | 244 (1.1) | 296 (1.4) | 151 (1.0) | 159 (1.1) | 15 (0.7) | 24 (0.9) |
| Anticholinergic agent | 1,671 (5.1) | 1,638 (7.6) | 928 (4.5) | 611 (3.8) | 1,302 (9.2) | 161 (7.6) | 66 (2.5) |
| Levodopa + MAOBI | 2,435 (7.4) | 1,448 (6.7) | 1,597 (7.7) | 870 (5.5) | 852 (6.0) | 108 (5.1) | 195 (7.3) |
| Levodopa + DA – non-ergot | 3,601 (10.9) | 2,336 (10.8) | 2,358 (11.3) | 1,383 (8.7) | 1,517 (10.7) | 207 (9.8%) | 238 (9.0%) |
| Levodopa + Zonisamide | 2,740 (8.3) | 1,752 (8.1) | 1,908 (9.2) | 1,329 (8.4) | 1,181 (8.3) | 195 (9.2) | 207 (7.8) |
| Levodopa + COMTI | 1,742 (5.3) | 1,173 (5.4) | 1,106 (5.3) | 923 (5.8) | 809 (5.7) | 172 (8.1) | 150 (5.6) |
| Levodopa + Droxidopa | 1,455 (4.4) | 922 (4.3) | 931 (4.5) | 873 (5.5) | 607 (4.3) | 100 (4.7) | 539 (20.3) |
| Levodopa + Amantadine | 1,660 (5.0) | 998 (4.6) | 1,033 (5.0) | 890 (5.6) | 697 (4.9) | 104 (4.9) | 100 (3.8) |
| Levodopa + Anticholinergic agent | 858 (2.6) | 573 (2.7) | 531 (2.6) | 348 (2.2) | 439 (3.1) | 64 (3.0) | 51 (1.9) |

COMTI, catechol-*O*-methyltransferase inhibitor; DA, dopamine agonist; MAOBI, monoamine oxidase type B inhibitor; PD, Parkinson’s disease; SD, standard deviation.

**SUPPLEMENTARY Table 6** Demographics and clinical characteristics of patients who newly initiated PD treatment and who stayed on levodopa monotherapy during the observation period or switched to adjunct therapy.

| **Characteristic** | **Levodopa**  **monotherapy**  ***N* = 1,398** | **Levodopa adjunct therapy****^†^**  ***N* = 1,092** | **Group Difference**  ***P*-value** |
| --- | --- | --- | --- |
| Male, *n* (%) | 607 (43.4) | 551 (50.5) | 0.001 |
| Age (years) | 76.4 ± 9.6 | 70.3 ± 10.9 | 0.000 |
| Age, *n* (%) |  |  |  |
| ≤54 years | 41 (2.9) | 96 (8.8) | 0.000 |
| 55–64 years | 128 (9.2) | 212 (19.4) | 0.000 |
| 65–74 years | 278 (19.9) | 305 (27.9) | 0.000 |
| 75–84 years | 690 (49.4) | 409 (37.5) | 0.000 |
| ≥85 years | 261 (18.7) | 70 (6.4) | 0.000 |
| Observation period (year) | 3.5 ± 0.9 | 3.9 ± 1.0 | 0.000 |
| Duration of PD treatment (days) | 545.0 ± 287.0 | 732.8 ± 364.6 | 0.000 |
| Charlson Comorbidity Index | 1.4 ± 1.7 | 1.1 ± 1.6 | 0.000 |
| Number of anti-PD drug types | 1.0 ± 0.1 | 2.5 ± 0.9 | 0.000 |
| Number of drugs by ATC (major category), | 2.5 ± 1.9 | 2.2 ± 1.8 | 0.000 |
| Comorbidities, *n* (%) |  |  |  |
| Constipation | 636 (45.5) | 397 (36.4) | 0.000 |
| Pain | 408 (29.2) | 288 (26.4) | 0.132 |
| Insomnia | 363 (26.0) | 214 (19.6) | 0.000 |
| Depression and anxiety | 231 (16.5) | 160 (14.7) | 0.223 |
| Cognitive disorder | 179 (12.8) | 51 (4.7) | 0.000 |
| Hallucination | 7 (0.5) | 8 (0.7) | 0.630 |

Data are mean ± SD unless otherwise stated.

^†^Patients who switched to monotherapy with other drug(s) were not included.

ATC, Anatomical Therapeutic Chemical; PD, Parkinson’s disease; SD, standard deviation.

**SUPPLEMENTARY Table 7** Factors associated with duration of levodopa monotherapy in patients who newly initiated PD treatment (*N* = 2,575).

| **Variable** | **Hazard ratio** | **95% CI** | ***p*-value** |
| --- | --- | --- | --- |
| Age (years) | 0.97 | 0.961–0.979 | 0.000 |
| Sex (ref. female) | 1.138 | 1.013–1.279 | 0.030 |
| Comorbidities^†^, middle category (ref. no comorbidity) | |  |  |
| Depression and anxiety | 0.929 | 0.784–1.102 | 0.399 |
| Hallucination | 2.048 | 1.018–4.122 | 0.045 |
| Cognitive disorder | 0.569 | 0.433–0.747 | 0.000 |
| Number of concomitant drugs^†^ | 1.010 | 0.994–1.027 | 0.223 |
| Charlson Comorbidity Index^†^ | 0.978 | 0.938–1.019 | 0.290 |
| Database (ref. JMDC database) |  |  |  |
| Elderly | 0.942 | 0.734–1.210 | 0.641 |
| NHI | 1.033 | 0.867–1.230 | 0.717 |

^†^Within 3 months of starting the second prescription pattern.

CI, confidence interval; NHI, National Health Insurance; PD, Parkinson’s disease; ref, reference.

**SUPPLEMENTARY Table** **8** Factors associated with a prescription for non-ergot DA monotherapy in patients who newly initiated PD treatment (*N* = 3,467).

| **Variable** | **Odds ratio** | **95% CI** | ***p*-value** |
| --- | --- | --- | --- |
| Age (years) | 0.971 | 0.955–0.987 | 0.001 |
| Sex (ref. female) | 0.812 | 0.624–1.054 | 0.118 |
| Comorbidities^†^, middle category (ref. no comorbidity) | |  |  |
| Depression and anxiety | 1.250 | 0.883–1.753 | 0.201 |
| Hallucination | 0.509 | 0.028–2.627 | 0.520 |
| Cognitive disorder | 0.642 | 0.296–1.234 | 0.219 |
| Number of concomitant drugs^†^ | 1.061 | 1.026–1.096 | 0.001 |
| Charlson Comorbidity Index^†^ | 0.994 | 0.903–1.086 | 0.895 |
| Database (ref. JMDC database) |  |  |  |
| Elderly | 0.335 | 0.197–0.570 | 0.000 |
| NHI | 0.710 | 0.497–1.010 | 0.058 |

^†^During3 months before the index date.

CI, confidence interval; DA, dopamine agonist; NHI, National Health Insurance; PD, Parkinson’s disease; ref, reference.

**SUPPLEMENTARY Table 9** Factors associated with duration of non-ergot DA monotherapy in patients who newly initiated PD treatment (*N* = 267).

| **Variable** | **Hazard ratio** | **95% CI** | ***p*-value** |
| --- | --- | --- | --- |
| Age (years) | 1.013 | 0.990–1.037 | 0.276 |
| Sex (ref. female) | 0.913 | 0.636–1.311 | 0.622 |
| Comorbidities^†^, middle category (ref. no comorbidity) | |  |  |
| Depression and anxiety | 0.730 | 0.423–1.258 | 0.257 |
| Hallucination | 0.000 | 0.000–∞ | 0.996 |
| Cognitive disorder | 0.616 | 0.147–2.577 | 0.507 |
| Number of concomitant drugs^†^ | 0.961 | 0.913–1.011 | 0.126 |
| Charlson Comorbidity Index^†^ | 0.861 | 0.726–1.021 | 0.085 |
| Database (ref. JMDC database) |  |  |  |
| Elderly | 0.518 | 0.224–1.199 | 0.125 |
| NHI | 0.939 | 0.586–1.502 | 0.792 |

^†^Within 3 months of starting the second prescription pattern.

CI, confidence interval; DA, dopamine agonist; ∞, infinity; NHI, National Health Insurance; PD, Parkinson’s disease; ref, reference.

**SUPPLEMENTARY Table 10** Demographics and clinical characteristics of elderly patients ≥75 years who newly initiated PD treatment and who stayed on levodopa monotherapy during the observation period or switched to levodopa and non-ergot DA adjunct therapy.

| **Characteristic** | **Levodopa**  **monotherapy**  ***N* = 951** | **DA adjunct therapy^†^**  ***N* = 96** | **Group Difference**  ***p*-value** |
| --- | --- | --- | --- |
| Male, *n* (%) | 385 (40.5) | 40 (41.7) | 0.908 |
| Age (years) | 81.8 ± 4.5 | 78.9 ± 3.5 | 0.000 |
| Age, *n* (%) |  |  |  |
| ≤54 years | NA | NA | NA |
| 55–64 years | NA | NA | NA |
| 65–74 years | NA | NA | NA |
| 75–84 years | 690 (72.6) | 88 (91.7) | 0.000 |
| ≥85 years | 261 (27.4) | 8 (8.3) | 0.000 |
| Observation period (year) | 3.2 ± 0.8 | 3.2 ± 0.7 | 0.256 |
| Duration of PD treatment (days) | 537.2 ± 272.2 | 657.3 ± 304.1 | 0.000 |
| Charlson Comorbidity Index | 1.6 ± 1.8 | 1.6 ± 2.1 | 0.209 |
| Number of anti-PD drug types | 1.0 ± 0.1 | 2.3 ± 0.6 | 0.000 |
| Number of drugs by ATC (major category) | 2.8 ± 1.9 | 2.8 ± 1.8 | 0.830 |
| Comorbidities, *n* (%) |  |  |  |
| Constipation | 514 (54.0) | 53 (55.2) | 0.912 |
| Pain | 323 (34.0) | 37 (38.5) | 0.431 |
| Insomnia | 289 (30.4) | 31 (32.3) | 0.788 |
| Depression and anxiety | 166 (17.5) | 20 (20.8) | 0.493 |
| Cognitive disorder | 157 (16.5) | 5 (5.2) | 0.006 |
| Hallucination | 5 (0.5) | 2 (2.1) | 0.129 |

Data are mean ± SD unless otherwise stated.

^†^Patients who switched to monotherapy with other drug(s) were not included.

ATC, Anatomical Therapeutic Chemical; DA, dopamine agonist; PD, Parkinson’s disease; SD, standard deviation.

**SUPPLEMENTARY Table 11** Demographics and clinical characteristics of patients who increased levodopa dose from <300 mg to ≥300 mg during the observation period.

| **Characteristic** | | **Total**  ***N* = 8,677** | **≥75 years *N* = 5,879** | | **<75 years**  ***N* = 2,798** | ***p*-value** |
| --- | --- | --- | --- | --- | --- | --- |
| Male, *n* (%) | | 3,890 (44.8) | 2,443 (41.6) | | 1,447 (51.7) | 0.000 |
| Age (years) | | 75.9 ± 10.5 | 81.8 ± 4.8 | | 63.5 ± 8.0 | 0.000 |
| Age, *n* (%) | |  |  | |  |  |
| ≤54 years | | 403 (4.6) | NA | | 403 (14.4) | NA |
| 55–64 years | | 880 (10.1) | NA | | 880 (31.5) | NA |
| 65–74 years | | 1,515 (17.5) | NA | | 1515 (54.1) | NA |
| 75–84 years | | 4,234 (48.8) | 4,234 (72.0) | | NA | NA |
| ≥85 years | | 1,645 (28.0) | 1,645 (28.0) | | NA | NA |
| Observation period (year) | | 3.3 ± 1.1 | 2.9 ± 0.9 | | 4.0 ± 1.2 | 0.000 |
| Duration of PD treatment (days) | | 580.9 ± 398.3 | 520.0 ± 346.1 | | 708.7 ± 465.0 | 0.000 |
| Duration from index date to levodopa prescription ≥300 mg (days) | | 307.1 ± 287.5 | 297.9 ± 267.3 | | 326.2 ± 325.1 | 0.612 |
| Charlson Comorbidity Index | | 2.0 ± 2.0 | 2.4 ± 2.1 | | 1.2 ± 1.6 | 0.000 |
| Number of anti-PD drug types | | 1.5 ± 0.9 | 1.4 ± 0.8 | | 1.7 ± 1.0 | 0.000 |
| Number of drugs, ATC major category | | 3.0 ± 1.8 | 3.3 ± 1.7 | | 2.2 ± 1.7 | 0.000 |
| Comorbidities, *n* (%) |  | | |  | |  |
| Constipation | | 5,376 (62.0) | 4,160 (70.8) | | 1,216 (43.5) | 0.000 |
| Insomnia | | 3,105 (35.8) | 2,431 (41.4) | | 674 (24.1) | 0.000 |
| Pain | | 2,973 (34.3) | 2,429 (41.3) | | 544 (19.4) | 0.000 |
| Cognitive disorder | | 2,235 (25.8) | 2,015 (34.3) | | 220 (7.9) | 0.000 |
| Depression and anxiety | | 2,089 (24.1) | 1,491 (25.4) | | 598 (21.4) | 0.000 |
| Hallucination | | 165 (1.9) | 137 (2.3) | | 28 (1.0) | 0.000 |

Data are mean ± SD unless otherwise stated.

ATC, Anatomical Therapeutic Chemical; NA, not applicable; PD, Parkinson’s disease; SD, standard deviation

**SUPPLEMENTARY FIGURE 1** Flow diagram.


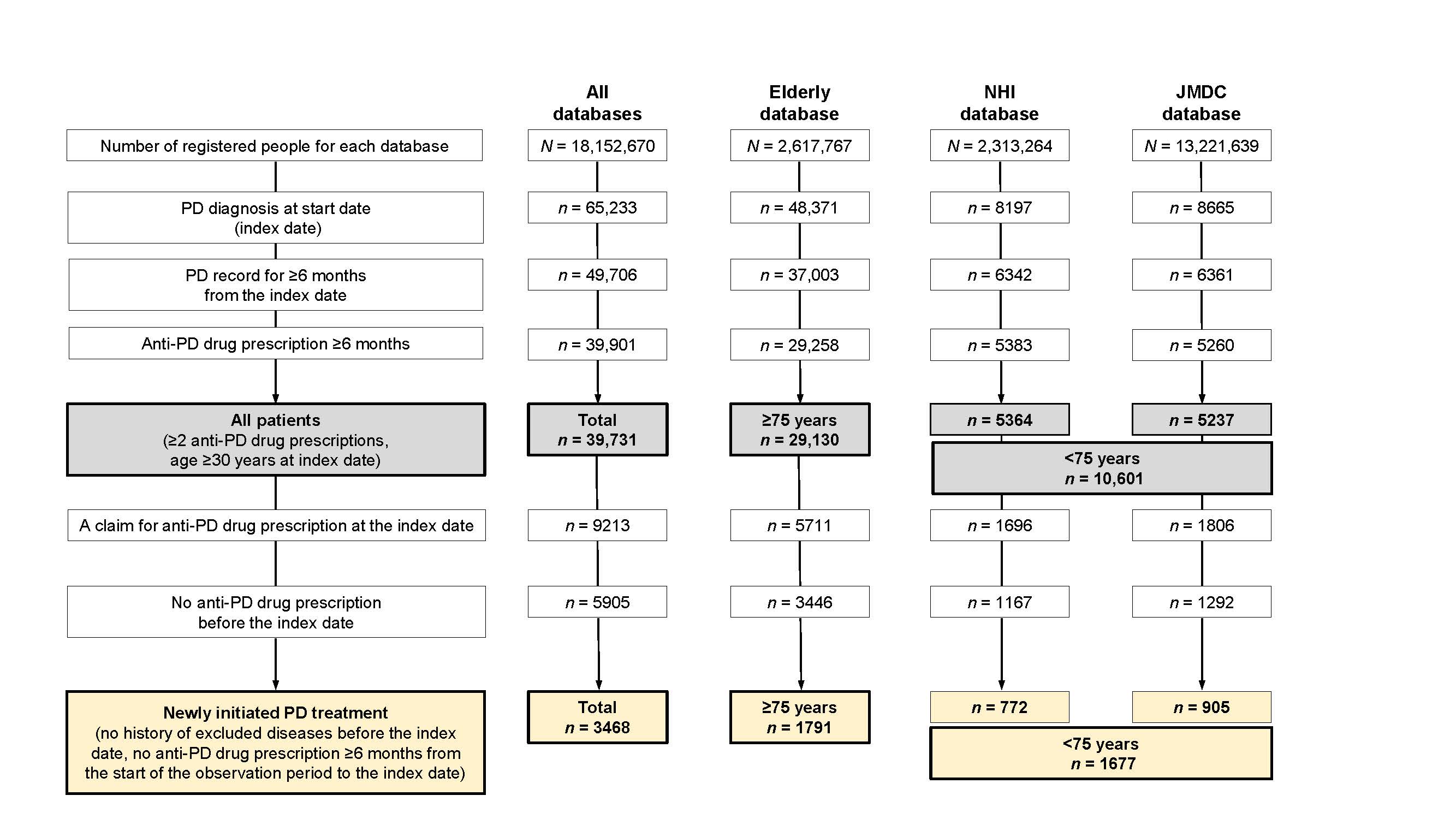


NHI, National Health Insurance; PD, Parkinson’s disease.

**SUPPLEMENTARY FIGURE 2** Maximum levodopa doses prescribed by age group during the observation period (All patients and those who newly initiated PD treatment).


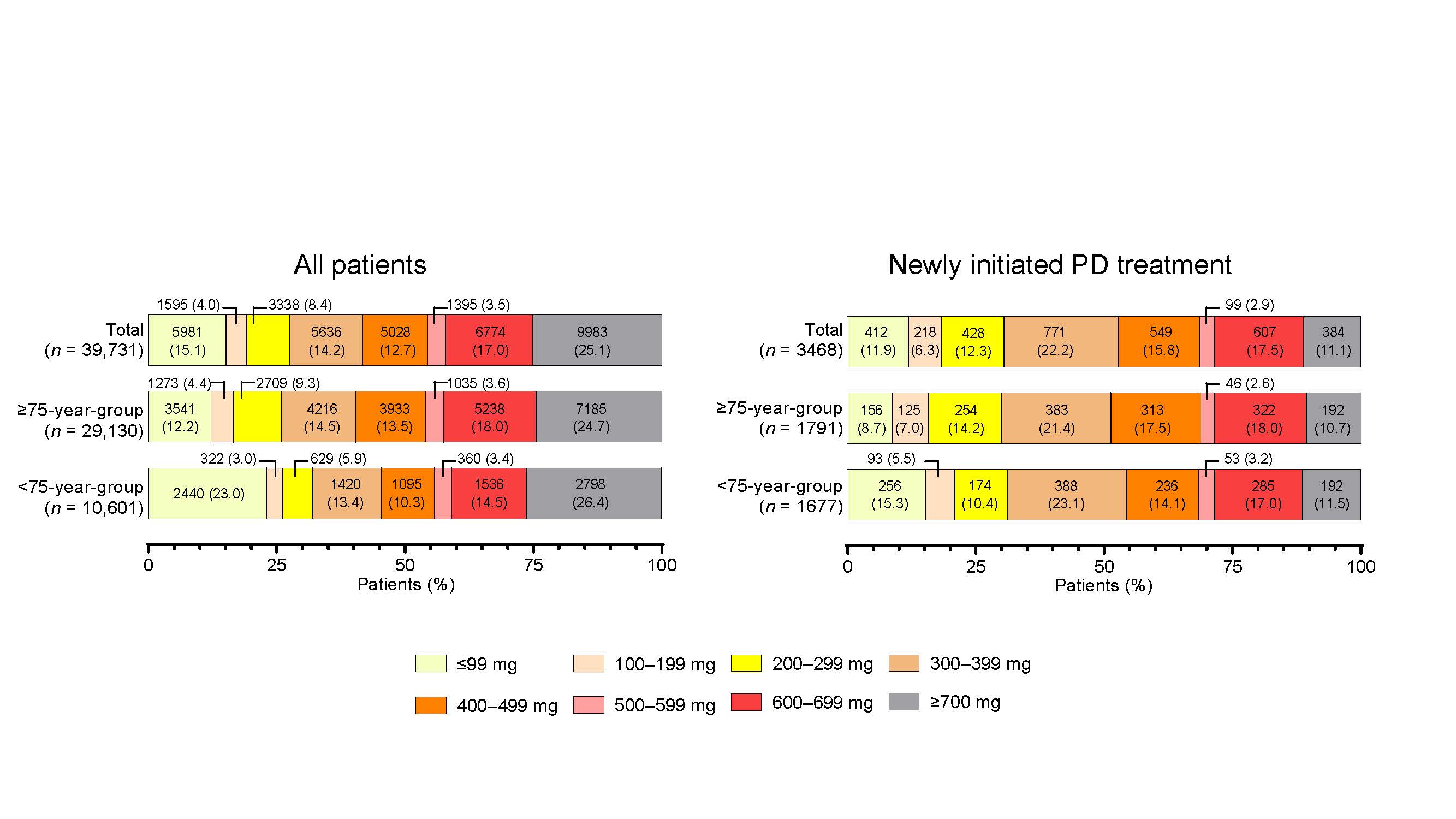


PD, Parkinson’s disease.

**SUPPLEMENTARY FIGURE 3** Longitudinal analysis of the type and combinations of anti-PD drugs prescribed during the observation period: Patients Total (*N* = 39,731), patients 65–74 years (*N* = 5,312), and patients ≥85 years (*N* = 8,162). The number of nodes and arrows in patients Total (11 and 15, respectively), 65–74 years (13 and 18, respectively), and ≥85 years (8 and 13, respectively).


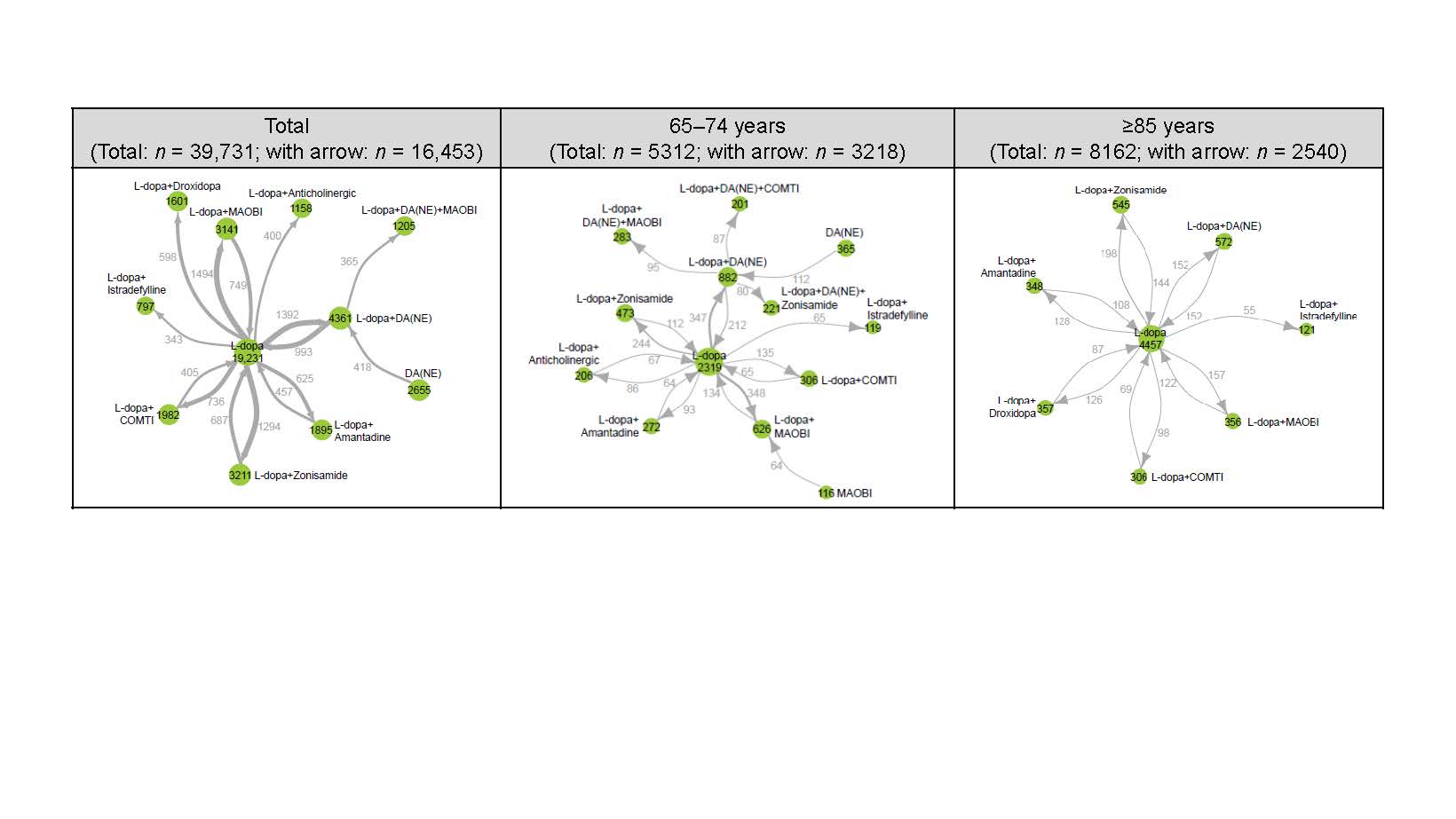
 COMTI, catechol-O-methyltransferase inhibitor; DA, dopamine agonist; L-dopa, levodopa; MAOBI, monoamine oxidase type B inhibitor; NE, non-ergot; PD, Parkinson’s disease.

**SUPPLEMENTARY FIGURE 4** Duration of non-ergot DA monotherapy in patients who newly initiated PD treatment with a first prescription for non-ergot DA monotherapy (*N* = 267).


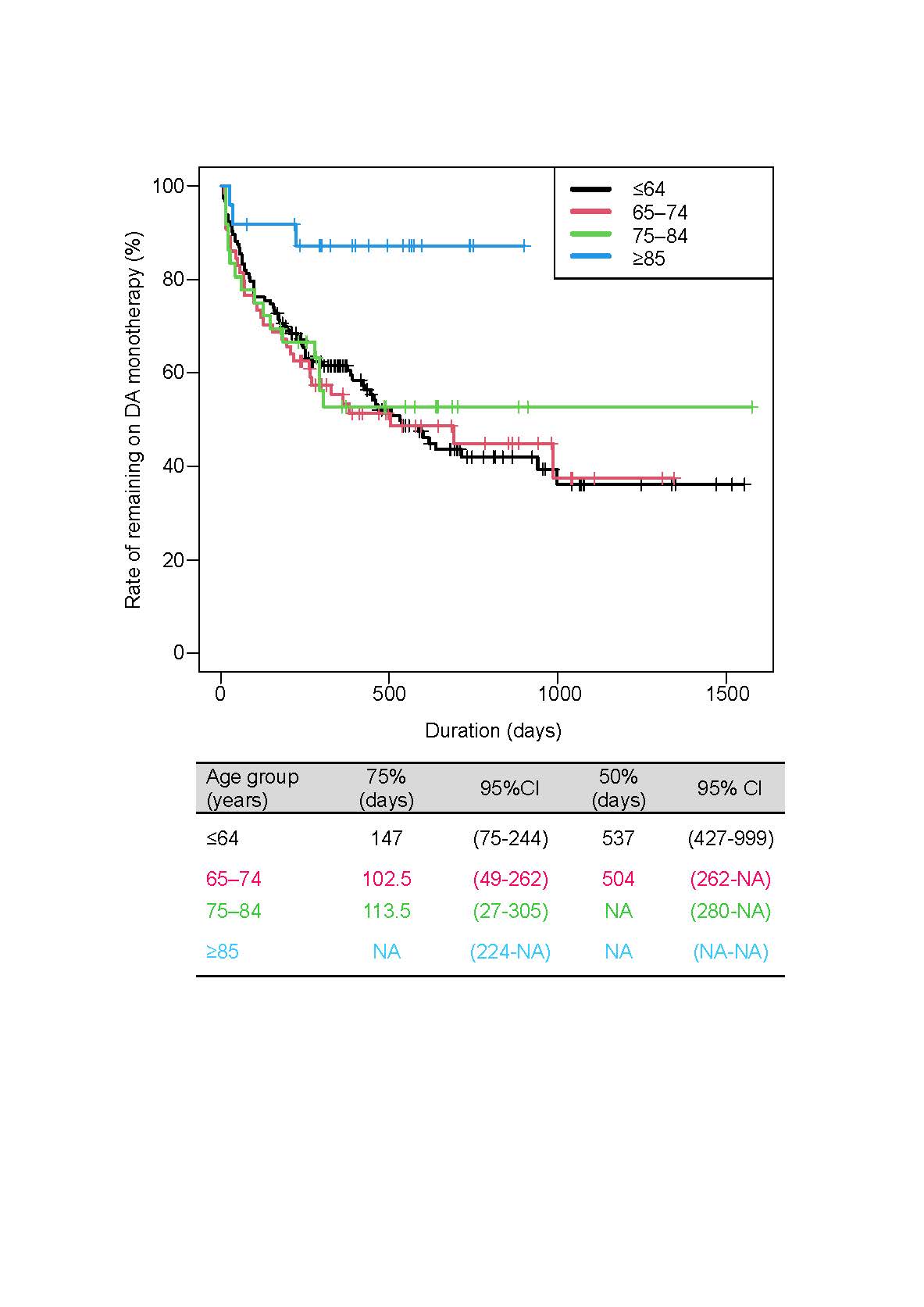


CI, confidence interval; DA, dopamine agonist; PD, Parkinson’s disease.

**SUPPLEMENTARY FIGURE 5** Treatment modalities and drug prescriptions when patients who increased their levodopa dose to ≥300 mg. (**A**) Total, (**B**) ≥75-year-group, and (**C**) <75-year-group.


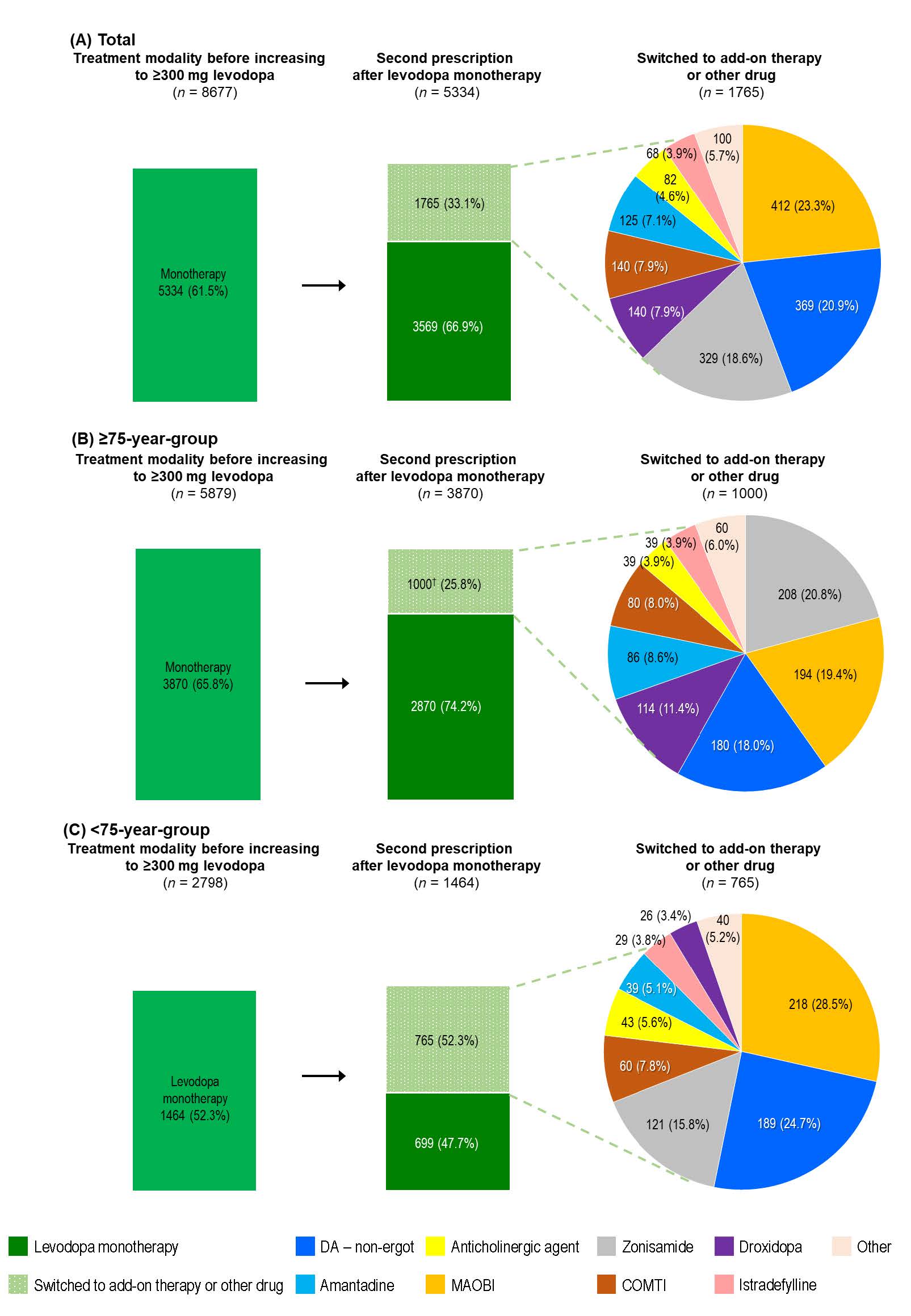
 COMTI, catechol-O-methyltransferase inhibitor; DA, dopamine agonist; MAOBI, monoamine oxidase type B inhibitor; PD, Parkinson’s disease.
